# Multiple calcified basal cell carcinomas in an asymptomatic patient with a very elevated coronary calcium score

**DOI:** 10.1016/j.jdcr.2025.10.016

**Published:** 2025-10-20

**Authors:** Jena C. Jacobs, Mario Sequeira

**Affiliations:** a Bassett Medical Center, Cooperstown, New York; b Brevard Skin and Cancer Center, Rockledge, Florida; c University of Miami Miller School of Medicine, Miami, Florida

**Keywords:** basal cell carcinoma, calcinosis, calcium, carcinoma, coronary artery calcification, coronary artery disease, Mohs micrographic surgery

## Introduction

Basal cell carcinoma (BCC) is the most common type of skin cancer, with 3.6 million cases diagnosed annually.^1^ BCC is typically caused by prolonged exposure to UV radiation, making it more common in sun-exposed areas.^1^ While BCC grows slowly and rarely metastasizes to other parts of the body, it can be locally invasive and cause significant damage to surrounding tissues if left untreated.^2^

Calcium is the most abundant mineral in the human body. Mostly found in teeth and bone, approximately 1% is dissolved in the bloodstream. As aging occurs, calcium is deposited throughout the body.^3^ The incidence of calcification in BCC is approximately 14%.^4^

It typically appears as small, irregular deposits within the tumor tissue and can result from necrosis, dystrophic calcification, or cellular degeneration within the tumor.^2^ Calcification is more commonly observed in other skin neoplasms, such as follicular-derived tumors.^2^ The presence of calcium in BCC has not been previously recognized as a feature of coronary calcification.

Coronary calcification, measured by a coronary artery calcium (CAC) score, refers specifically to calcium deposits within coronary arteries. The presence of coronary calcification is universal in all patients with documented coronary artery disease (CAD) and occurs through entirely different pathophysiological mechanisms than calcification observed in skin tumors.^3^^,^^5^ The CAC score quantifies calcium in coronary arteries, a marker of atherosclerosis and CAD risk.^3^ The scoring scale provides risk stratification: 0 denotes no detectable calcium and minimal CAD risk; scores between 1 and 10 reflect low calcium presence and slight CAD risk; scores of 11-99 indicate mild calcification with moderate CAD risk; scores of 100-399 denote moderate calcium levels with increased CAD risk; and scores of 400 or higher signify extensive calcification, marking a high CAD risk and often necessitating aggressive intervention.^3^ Severe coronary calcification (score >1000) is associated with advanced obstructive coronary disease.^3^ In the case of our patient, his CAC score was 1,265, predominantly concentrated in the left anterior descending artery, indicative of severe coronary calcification and justifying pharmacologic management to mitigate disease progression.

## Case report

**Fig 1:**
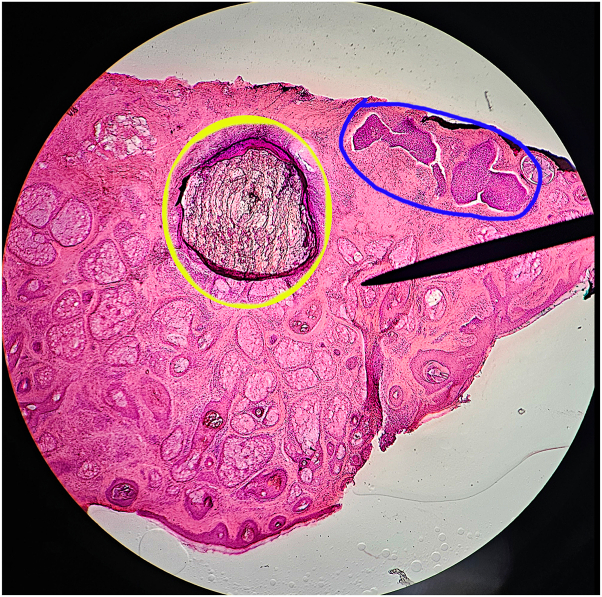
Histologic correlation of dermal calcification and basal cell carcinoma in a biopsy specimen from the left forehead on January 2, 2018. H&E-stained section showing a focus of dermal calcification (denoted by *yellow circle*) in proximity to areas of basal cell carcinoma (denoted by *blue circle*). *H&E*, Hematoxylin and eosin.

**Fig 2:**
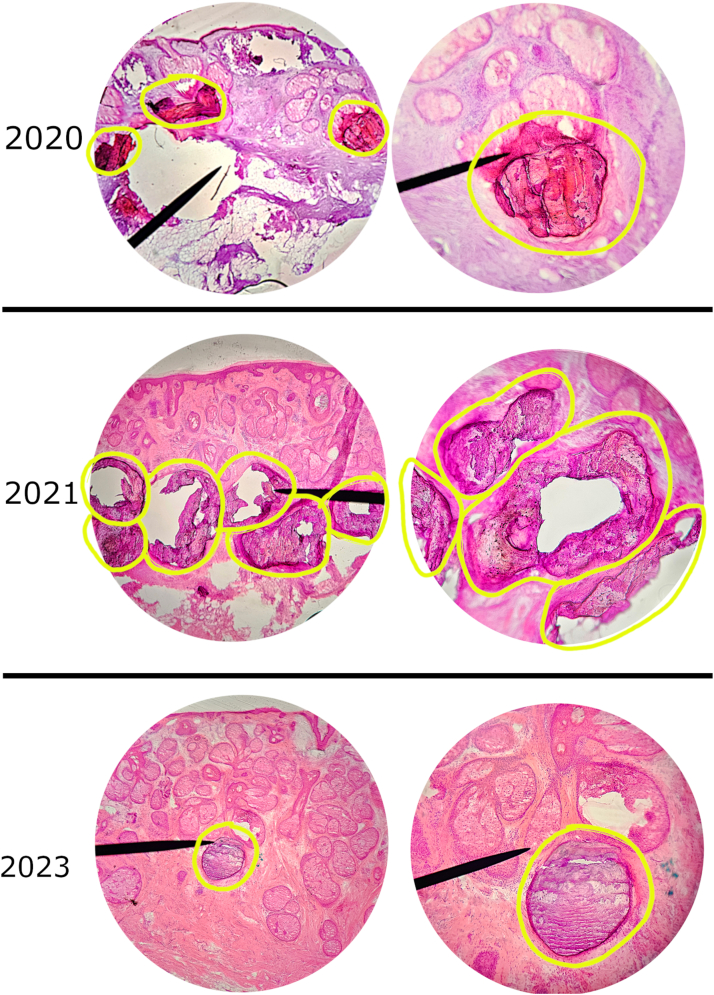
Areas of dermal calcium deposition, denoted by *yellow circles*, identified during Mohs micrographic surgery for basal cell carcinoma at 3 distinct anatomical sites: August 18, 2020, right superior temple; March 30, 2021, mid forehead; and August 18, 2023, right cheek. Representative hematoxylin and eosin (H&E)-stained sections are shown at 10× and 40× magnification, respectively.

A 78-year-old White male presented to our cancer center from 2018 to 2023 with multiple BCCs, all previously treated successfully with Mohs micrographic surgery. Upon microscopic review of the tissue specimens excised in previous years, dystrophic calcification was noted in the dermis, both adjacent to the tumor and, in some cases, even when the tumor was cleared and no longer present in additional deeper Mohs sections (Figs 1 and 2). Interestingly, several histologic subtypes of BCCs were calcified (Table I).

**Table I:** Calcified histological subtypes of BCC in the patient

Date	Location	Histological subtypes
November 5, 2024	Right jawline	Nodular BCC with squamoid differentiation
August 18, 2023	Right cheek	Nodular BCC
November 3, 2021	Right cheek	Nodular BCC
March 30, 2021	Left mid-forehead	Infiltrative BCC with squamoid differentiation
August 18, 2020	Right temple	Nodular BCC
January 2, 2018	Left superior forehead	Nodular BCC

*BCC*, Basal cell carcinoma.

These findings raised the question of a potentially systemic calcific process; however, the patient denied symptoms of peripheral vascular disease, CAD, or medical conditions associated with calcinosis cutis. In the interest of precaution, the patient had an initial serum calcium ordered on May 26, 2021, which was normal. The patient was then referred to cardiology for a coronary calcium score, which revealed a significantly elevated score of 1265.

His relevant medical history included nonmelanoma skin cancer, hypertension, and hyperlipidemia with prior statin intolerance to atorvastatin due to lower extremity weakness. Additional medications included aspirin 81 mg every other day, lisinopril 20 mg daily, and supplements such as CoQ-10 (10 mg daily), fish oil, meloxicam (7.5 mg as needed), and SAM-e (400 mg daily). He denied taking calcium- or phosphate-containing agents. Notable drug allergies included sulfonamide antibiotics.

Family history was significant for cardiovascular disease in his mother and diabetes in his brother. Although the patient was asymptomatic for symptoms such as chest pain, shortness of breath, and claudication, he reported recent decreases in activity level. He has a body mass index of 26.5, no history of tobacco use, and reported moderate alcohol consumption (2-5 drinks per week). Physical examination findings were unremarkable. Lab work revealed normal blood calcium (9.8 mg/dl), phosphorus (2.9 mg/dl), and triglycerides (117 mg/dl), but elevated cholesterol (232 mg/dl, low-density lipoprotein [LDL] 159 mg/dl). An electrocardiogram and nuclear stress test were normal. A 2D echocardiogram showed normal left ventricular function with mild mitral and tricuspid regurgitation.

Recommended management included aggressive risk factor modification, pitavastatin (Livalo), initially prescribed at 2 mg daily and increased to 4 mg due to inadequate LDL cholesterol control, and the addition of ezetimibe 10 mg daily. His LDL, previously at 159 mg/dL, improved to 91 mg/dL with the dosage adjustment, though his target was not in the desired range (below 70 mg/dL).

## Discussion

Recent studies have reported a direct correlation between low calcium deposition in the bone and its parallel accumulation in the blood vessel wall or so called the “calcium paradox,” partially explaining the association between vascular calcification and risk of bone fractures.^6^ The presence of dermal calcification within or surrounding BCC lesions is considered incidental. Although there is a positive association between CAC score and serum calcium, not all studies have found a significant correlation to recommend serum calcium as a screening test.^7^^,^^8^ In this case, the repeated intraoperative identification of calcification during Mohs micrographic surgery prompted further evaluation for systemic disease, which revealed a markedly elevated CAC score exceeding 1000, well above the established threshold for high-risk CAD despite being asymptomatic. The temporal association between repeated calcified BCCs and significant vascular calcification raises the question of whether dermal calcification could, in select cases, reflect underlying systemic pathology. While causality cannot be inferred, this observation may warrant referral for a cardiovascular assessment by dermatopathologists and Mohs surgeons encountering patients with multiple or recurrent calcified BCCs, particularly those with known cardiovascular risk factors. If corroborated in larger studies, this association could offer a novel route for early detection of CAD in dermatology cancer patients.

## Conflicts of interest

None disclosed.
